# Derivation and Validation of a Score Using Prehospital Data to Identify Adults With Trauma Requiring Early Laparotomy

**DOI:** 10.1001/jamanetworkopen.2021.45860

**Published:** 2022-01-31

**Authors:** Adam Gutierrez, Kazuhide Matsushima, Areg Grigorian, Morgan Schellenberg, Kenji Inaba

**Affiliations:** 1 Division of Acute Care Surgery, University of Southern California, Los Angeles, California

## Abstract

**Importance:**

A scoring tool to identify which adults with traumatic injury will require early laparotomy could help improve prehospital triage and system readiness.

**Objective:**

To develop and validate a prediction model using prehospital information for early laparotomy following trauma.

**Design, Setting, and Participants:**

This retrospective cohort study analyzed data from the 2017 version of the American College of Surgeons Trauma Quality Improvement Program database. All adult patients with traumatic injury aged 18 years or older who were admitted to a US trauma center in 2017 were included. Patients were randomly assigned to a derivation or validation cohort. Data were collected and analyzed between July 2020 and September 2020.

**Main Outcomes and Measures:**

The primary outcome was laparotomy within 2 hours of hospital arrival. A scoring system was developed to predict early laparotomy using a logistic regression model in the derivation cohort. This was validated in the validation cohort using the area under the receiver operating characteristic curve.

**Results:**

A total of 379 890 US adults with traumatic injury were included; 190 264 patients were in the derivation cohort and 189 626 patients were in the validation cohort. The cohorts had the same proportion of laparotomy within 2 hours of hospital arrival (1.1%). The median (IQR) age was 32 (25-46) years in the early laparotomy group and 54 (33-72) years in the group with no early laparotomy. The early laparotomy group contained 113 776 of 188 211 (60.5%) male patients, while the group with no early laparotomy contained 1702 of 2053 (82.9%) male patients. The variable most strongly associated with early laparotomy was penetrating injury to the head, neck, torso, or extremities proximal to the elbow or knee (odds ratio, 13.47; 95% CI, 12.22-14.86) with a point value of 10 (maximum overall score 20). Other variables included in the scoring system were the male sex, a systolic blood pressure less than 90 mm Hg, a Glasgow Coma Scale of less than or equal to 13, having chest wall instability or deformity, pelvic fracture, and high-risk blunt mechanism. In the validation cohort, the C statistic of the scoring system was 0.78 (95% CI, 0.77-0.79).

**Conclusions and Relevance:**

In this study, a novel scoring tool using prehospital information was derived and validated to identify which adults with traumatic injury will require laparotomy within 2 hours of hospital arrival. This tool may help trauma professionals allocate operative team resources before patient arrival.

## Key Points

**Question:**

Can patients requiring early laparotomy following trauma be identified using prehospital information?

**Findings:**

In this cohort study including 379 890 adults with traumatic injury, a model using the variables of sex, prehospital systolic blood pressure, Glasgow Coma Scale score, mechanism of injury, chest wall instability or deformity, and pelvic fracture successfully identified patients requiring early laparotomy.

**Meaning:**

These findings suggest that this model can be useful to identify the need for early laparotomy in patients with traumatic injury and triage them to appropriate hospitals.

## Teaser

This cohort study analyzes data from the American College of Surgeons Trauma Quality Improvement Program database to develop and validate a model to identify patients requiring early laparotomy following trauma.

## Introduction

Emergent laparotomy is a life-saving intervention in severely injured patients with intra-abdominal hemorrhage. Despite recent advances in trauma care, hemorrhage remains a major cause of trauma death, and many of these deaths are preventable.^1^ One study of 2594 trauma deaths found that the most common error contributing to death in patients with an initial low-to-moderate mortality risk was delayed operative control of intra-abdominal or pelvic hemorrhage.^2^ A delay in operative intervention or an embolization procedure for hypotensive patients with major torso trauma has indeed been shown to be associated with increased mortality.^3,4^ Every 1-hour delay in hemorrhage control may increase the risk of mortality by 50%.^5^ Many efforts in both the prehospital and emergency department setting have been instituted to mitigate the risk of delays in definitive management. In the prehospital setting, several initiatives, such as the development of trauma center triage criteria by the American College of Surgeons (ACS) Committee on Trauma, have addressed the issue of delays in care for adults with traumatic injury.

Prehospital information about an injured patient is often limited but can be critical to help clinicians in the hospital setting prepare to deliver the appropriate care in an expeditious fashion. Despite improvements in prehospital care and standardization of trauma center triage criteria, it remains challenging to identify which patients will require early operative intervention. There is currently no widely used prehospital stratification tool to predict which adults with traumatic injury will require laparotomy soon after arrival, which may help with hospital system readiness and resource allocation. For this reason, we sought to develop and validate a novel Prehospital Preparation for Surgery (PREPS) score using prehospital variables to identify the need for early laparotomy following trauma. We hypothesized that penetrating injuries to the torso would be most strongly associated with the need for early laparotomy.

## Methods

### Study Design and Data Source

This retrospective cohort study was approved by the institutional review board of the University of Southern California. A waiver of consent was granted given the use of deidentified data. The study followed the Strengthening the Reporting of Observational Studies in Epidemiology (STROBE) reporting guideline for observational studies.

All data were obtained from the ACS Trauma Quality Improvement Program (TQIP). TQIP was created in 2008 by the ACS Committee on Trauma with the intent of reducing variability in adult trauma outcomes and offering best practice guidelines for improved trauma care. The TQIP uses the National Trauma Data Standard (NTDS), which is a data set defining standardized data elements collected by the ACS.^6^ We included adult adults with traumatic injury 18 years of age or older from the 2017 version of the database because this was the first year in which all participating trauma centers universally started using *International Statistical Classification of Diseases and Related Health Problems, Tenth Revision *(*ICD-10*), which allowed for generalizability between centers. Additionally, this was the first year in which more granular prehospital data including trauma center criteria were available. We excluded all patients who were transferred from another hospital, as well as those with unknown trauma center triage criteria.

### Data Collection and Outcome Measures

All variables examined are prehospital data maintained in the TQIP. These variables include patient demographics, mechanism of injury, incident details, the first set of vital signs in the field, and trauma center criteria. Trauma center criteria included those based on mechanism as well as those based on injury (Box). The primary outcome was early laparotomy defined by those undergoing laparotomy within 2 hours of arrival with the following *ICD-10* codes: 0DJ00ZZ, 0DJ60ZZ, 0DJD0ZZ, 0DJU0ZZ, 0DJW0ZZ, 0WJG0ZZ, 0WJJ0ZZ, 0WJP0ZZ, and 0WJR0ZZ. Other outcomes included hospital length of stay, intensive care unit (ICU) length of stay, ventilator-days, and in-hospital mortality.

Box. **Criteria for Transport to a Trauma Center as Defined by the American College of Surgeons Committee on Trauma and the US Centers for Disease Control and Prevention**

**Physiologic and Anatomic Criteria**

Glasgow Coma Score ≤13
Systolic blood pressure <90 mm Hg
Respiratory rate <10 or >29 breaths per minute or need for ventilatory support
All penetrating injuries to the head, neck, torso, and extremities proximal to elbow or knee
Chest wall instability or deformity (eg, flail chest)
Two or more proximal long bone fractures
Crushed, degloved, mangled, or pulseless extremity
Amputation proximal to wrist or ankle
Pelvic fracture
Open or depressed skull fracture
Paralysis

**Mechanism of Injury Criteria**

Fall >20 ft
Crash intrusion, including roof: >12 in occupant site or >18 in any site
Crash ejection (partial or complete) from automobile
Crash death in same passenger compartment
Crash vehicle telemetry data consistent with high-risk injury
Automobile vs pedestrian/bicyclist thrown, run over, or >20 MPH impact
Motorcycle crash >20 MPH
Pregnancy >20 weeks
Burns
Burns with trauma
EMS professionals judgement

Abbreviations: EMS, emergency medical services; MPH, miles per hour.

### Statistical Analysis

We first randomized all entries in the TQIP data set and used half of the patients to derive PREPS and the other half to validate PREPS. The randomization was accomplished using the Markov chain approach.^7^ In the derivation set, PREPS was created using a 3-step method. Prehospital variables associated with early laparotomy were identified using a univariate analysis. These were chosen based on consensus among the study authors. We only selected variables that were reported by emergency medical technicians at the scene. High-risk blunt mechanism was defined by crash intrusion (including roof) from more than 12 inches from occupant site or more than 18 inches from any site, partial or complete crash ejection from automobile, or a pedestrian or bicyclist being hit by an automobile and thrown or run over or sustaining more than 20 miles per hour of impact. For continuous or discrete variables, a student *t* test was used. For categorical variables, a Fisher exact test or χ^2^ analysis was used as appropriate. Variables with *P* < .20 were then included in a multivariable forward stepwise logistic regression model to identify independent risk factors for early laparotomy using *P* < .05 as the cutoff for statistical significance. The relative effect of each risk factor was then used to derive a PREPS score. This was done by using the coefficient, dividing by the lowest common denominator, and rounding to the nearest integer to develop a point allocation for each risk factor. The area under receiver operating characteristic (AUROC) curve was examined to ensure consistency in the C statistic for both derivation and validation sets. PREPS was then validated in the validation cohort using the AUROC curve. This method to derive and validate a novel scoring tool has previously been performed in the literature.^8,9,10^ All analyses were performed with IBM SPSS Statistics for Windows version 24 (IBM Corp). Data were collected and analyzed between July 2020 and September 2020.

## Results

### Patient Characteristics

Of 379 890 study patients, there was an overall early laparotomy rate of 1.1%. The median (IQR) age was 32 (25-46) years in the early laparotomy group and 54 (33-72) years in the group with no early laparotomy. The early laparotomy group contained 113 776 of 188 211 (60.5%) male patients, while the group with no early laparotomy contained 1702 of 2053 (82.9%) male patients. There were no statistically significant differences between the derivation and validation sets in patient characteristics and injury profiles (eTable in the Supplement). In the derivation cohort, a total of 2053 patients required early laparotomy (1.1%). Table 1 shows a comparison of prehospital variables between patients who underwent early laparotomy and those who did not among the derivation cohort. Patients in the early laparotomy group were more likely to be male and younger. In addition, there were significant differences between these 2 groups for various prehospital variables, including vital signs in the field, mechanism of injury, and specific injury patterns. In the early laparotomy group, the in-hospital mortality was significantly higher (414 [20.2%] vs 6934 [3.7%], *P* < .001) and hospital length of stay was significantly longer (median [IQR], 7 [4-13] days vs 3 [2-6] days; *P* < .001).

**Table 1.** Patient Characteristics and Injury Profiles in the Early Laparotomy and No Early Laparotomy Groups Among the Derivation Cohort

Characteristic	No. (%)		*P* value
	No early laparotomy (n = 188 211)	Early laparotomy (n = 2053)	
Age, median (IQR)	54 (33-72)	32 (25-46)	<.001
Female sex	74 435 (39.5)	351 (17.1)	
Male sex	113 776 (60.5)	1702 (82.9)	<.001
Comorbidities			<.001
Congestive heart failure	6696 (3.6)	14 (0.7)	<.001
Cirrhosis	1631 (0.9)	18 (0.9)	.96
COPD	12 483 (6.6)	37 (1.8)	<.001
Diabetes	25 311 (13.4)	82 (4.0)	<.001
Hypertension	61 395 (32.6)	216 (10.5)	<.001
Smoking	37 608 (20.0)	510 (24.8)	<.001
Anticoagulant use	3792 (2.0)	7 (0.3)	<.001
End stage kidney disease	3162 (1.7)	4 (0.2)	<.001
ISS, median (IQR)	6 (4-10)	17 (9-27)	<.001
Trauma center criteria			
Prehospital			
GCS ≤ 13	11 735 (6.2)	299 (14.6)	<.001
SBP < 90	2941 (1.6)	201 (9.8)	<.001
RR < 10 or >29	2592 (1.4)	124 (6.0)	<.001
Penetrating injuries	8014 (4.3)	845 (41.2)	<.001
Proximal long bone fractures	1651 (0.9)	31 (1.5)	.002
Chest wall instability or deformity	544 (0.3)	21 (1.0)	<.001
Crush injury	1051 (0.6)	18 (0.9)	.06
Amputation	244 (0.1)	8 (0.4)	.001
Pelvic fracture	1149 (0.6)	27 (1.3)	<.001
Open or depressed skull fracture	720 (0.4)	15 (0.7)	.01
Paralysis	714 (0.4)	7 (0.3)	.78
High-risk blunt mechanism	12 687 (6.7)	235 (11.4)	<.001

Abbreviations: COPD, chronic obstructive pulmonary disease; GCS, Glasgow Coma Scale; ISS, injury severity score; RR, respiratory rate; SBP, systolic blood pressure.

### Model Development and Validation

In the multivariate logistic regression analysis, a total of 7 factors independently associated with for early laparotomy were identified to develop the PREPS scoring tool ranging from 0 to 20 (Table 2). The strongest predictor of early laparotomy was penetrating injury to the head, neck, torso, or extremities proximal to the elbow or knee (odds ratio, 13.47, 95% CI, 12.22-14.86) with an assigned point value of 10. The C statistic for the derivation and validation sets were 0.79 (95% CI, 0.77-0.80) and 0.78 (95% CI, 0.77-0.79), respectively (Figure).

**Table 2.** Univariate and Multivariable Logistic Regression Analysis for Early Laparotomy and PREPS Scoring System

Variable	OR (95% CI)		*P* value	Assigned points
	Crude	Adjusted		
Male sex	3.17 (2.83-3.56)	2.14 (1.90-2.40)	<.001	2
High risk blunt mechanism	1.79 (1.56-2.05)	2.34 (2.02-2.72)	<.001	2
Glasgow Coma Scale score ≤13	2.56 (2.27-2.90)	1.34 (1.16-1.55)	<.001	1
Systolic blood pressure <90 mm Hg	6.84 (5.89-7.94)	2.53 (2.13-3.02)	<.001	2
Penetrating injury to head, neck, torso, or extremities proximal to elbow or knee	15.73 (13.47-17.22)	13.47 (12.22-14.86)	<.001	10
Chest wall instability or deformity	3.57 (2.30-5.53)	2.17 (1.36-3.45)	.001	2
Pelvic fracture	2.17 (1.48-3.19)	1.94 (1.30-2.90)	.001	1
Maximum score	NA	NA	NA	20

Abbreviations: NA, not applicable; OR, odds ratio; PREPS, prehospital preparation for surgery.

**Figure.**
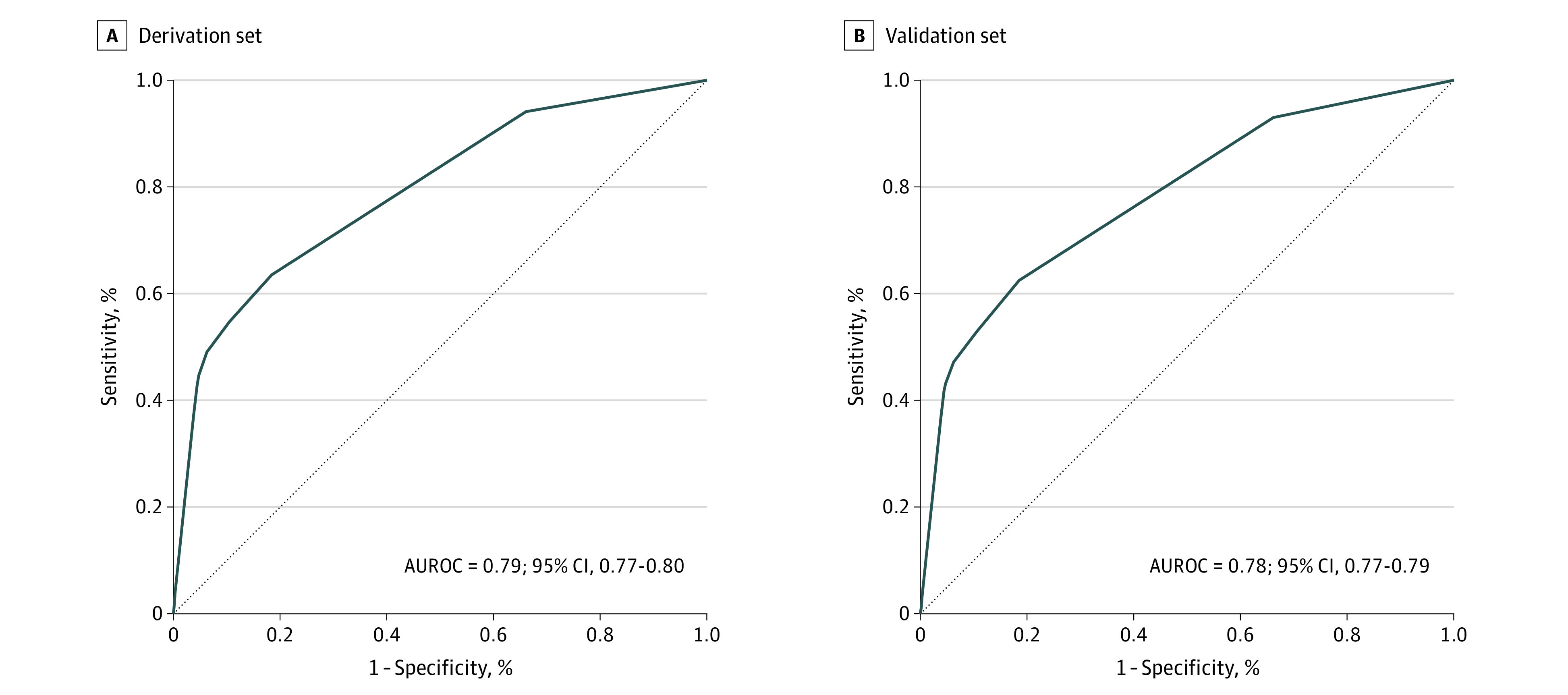
AUROC Curve for Development of the PREPS Score Panel A shows the derivation set: AUROC = 0.79; 95% CI, 0.77-0.80; panel B shows the validation set: AUROC = 0.78; 95% CI, 0.77-0.79. AUROC indicates area under the receiver operating characteristics; PREPS, prehospital preparation for surgery.

## Discussion

Using a nationwide database in the present study, a novel PREPS scoring tool was developed with only prehospital variables to identify the need for laparotomy within 2 hours of hospital arrival following trauma. Our results suggest that it would appear reasonable to anticipate the need for early laparotomy in patients with certain clinical factors related to patient demographics, mechanism of injury, and prehospital vital signs. Of those prehospital variables included in the PREPS score, the highest point was assigned to those with a penetrating injury to head, neck, torso, or extremities proximal to elbow or knee. This high point value for penetrating mechanism of injury is consistent with historical series demonstrating high rates of intra-abdominal injury from stab wounds and gunshot wounds.^11,12^ A review^13^ of over 11 000 patients with gunshot wounds showed that 53% of patients required an operation, however, the time to operation and specific type of operation was not specified. A different study focusing specifically on the need for laparotomy after penetrating abdominal trauma showed a laparotomy rate of 63%. Among those patients requiring laparotomy 95% underwent immediate laparotomy.^14^ This data from previous studies supports the strong predictive ability of penetrating injury for early laparotomy and justifies its high point allocation in our scoring tool.

It is well established that the use of prehospital information and tiered-trauma activation systems facilitate appropriate triage to trauma centers and promote efficient use of resources.^15^ With this in mind, implementation of the PREPS score has the potential to facilitate alterations in location of triage or resources available on arrival, such as an operating room or blood bank. Previous studies have examined using prehospital variables to predict the need for massive transfusion and other resuscitative care, but none have focused specifically on the need for early laparotomy.^16^ A scoring system based on ultrasonography findings in the emergency department has been created to predict which patients may require laparotomy after trauma, but such modalities are currently not available in the prehospital setting.^17^ Because of a lack of clinical tools for identifying patients requiring early laparotomy, significant delays in activating relevant teams and allocating resources are often associated with poor patient outcomes. A study by Clarke et al^3^ showed the probability of death increased approximately 1% for every 3 minutes a patient with traumatic injury was in the ED up to 90 minutes prior to undergoing laparotomy. A more recent study by Meizoso et al^4^ demonstrated an increased risk of mortality by almost 3-fold in hypotensive patients with traumatic injury with gunshot wounds who had delays of more than 10 minutes to the operating room. Therefore, implementation of the PREPS score also has the potential to improve patient outcomes, but further study is needed in this regard.

We believe that the PREPS scoring tool has several strengths. First, the score uses only prehospital information that is routinely collected by emergency medical services at the scene of an incident and does not require advanced equipment or diagnostic modalities. Second, in comparison to other trauma scoring systems, such as the Trauma and Injury Severity Score (TRISS), PREPS uses only binary variables.^18^ This allows for relative ease of calculation of the PREPS score in the field, and it can be relayed to in-hospital clinicians before patient arrival. Lastly, the scoring model was validated with a validation cohort of nearly 190 000 patients submitted from trauma centers across the US suggesting that it is generalizable.

### Limitations

There are several limitations to this study. First, the retrospective design of the study has inherent limitations, and the use of a nationwide database relies on accurate reporting and documentation of data in the medical record and the database itself. In the TQIP, for patients undergoing their laparotomy between 60 and 119 minutes, the timing of laparotomy was uniformly coded as 2 hours. Therefore, we defined early laparotomy as less than 2 hours in this study while various cutoffs have been used to define the golden hour following trauma in previous literature.^3,19^ There are also limitations related to the use of prehospital data. Some factors, such as those required to determine the presence of a high-risk blunt mechanism (ie, crash intrusion distance or passenger ejection), may be difficult or even dangerous to accurately assess at the scene of an incident due to environmental factors. There is also an inherent difficulty in identifying pelvic fractures in the prehospital setting without diagnostic imaging, save for obvious open fractures. The presence of a pelvic fracture is often determined by EMS personnel based on the mechanism of injury and physical examination findings in the field. A study by Lerner et al^20^ found that EMS perceived pelvic fracture was the least reliable indicator of the need for transport to a trauma center among the anatomic step of field triage guidelines.

Previous recommendations from the US Centers for Disease Control and Prevention proposed changing the language of the pelvic fracture criteria to pelvic instability or suspected pelvic fracture, but that was avoided for the sake of simplicity and to avoid an increase in over triage.^21^ In addition, SBP and GCS are dynamic variables that can be affected by pain, substance use, among other factors, which may make single data points unreliable. It is worth noting that the penetrating injury variable includes injury to the head, neck, and extremities, while our scoring tool is designed to identify only the need for early laparotomy and not any other surgical interventions. The significance of isolated penetrating injuries to the head, neck, and extremities and their effect on the scoring tool is unclear as these are grouped together with penetrating injuries to the torso. Given that this scoring tool uses only prehospital data, it is not intended to be the sole factor influencing the need for early laparotomy, but rather a prehospital adjunct to thorough assessment in the trauma bay. Our hope in designing this scoring tool was to serve as an early indicator to trauma clinicians of the potential need for operative intervention in adults with traumatic injury and facilitate system readiness.

## Conclusions

The PREPS score is the first tool of its kind, to our knowledge, that may help trauma clinicians allocate operative team resources before patient arrival. Further studies are needed to prospectively assess our scoring tool and elucidate its utility in the clinical setting.

## Notes

Supplement. **eTable. **Patient Characteristics in the Derivation and Validation Cohorts
