# Supplementary material for: Derivation and Validation of a Score Using Prehospital Data to Identify Adults With Trauma Requiring Early Laparotomy
Source: JAMA Netw Open. 2022 Jan 31;5(1):e2145860. doi: 10.1001/jamanetworkopen.2021.45860 (PMC8804917; doi:10.1001/jamanetworkopen.2021.45860)
Supplement: Supplement. — eTable. Patient Characteristics in the Derivation and Validation Cohorts [file jamanetwopen-e2145860-s001.pdf]

Supplemental Online Content

Gutierrez A, Matsushima K, Grigorian A, Schellenberg M, Inaba K. Derivation and validation of a score using prehospital data to identify adults with trauma requiring early laparotomy. *JAMA Netw Open*. 2022;5(1):e2145860. doi:10.1001/jamanetworkopen.2021.45860

eTable. Patient Characteristics in the Derivation and Validation Cohorts

This supplemental material has been provided by the authors to give readers additional information about their work.

© 2022 Gutierrez A et al. *JAMA Network Open.*

eTable. Patient Characteristics in the Derivation and Validation Cohorts

**Characteristic**	**Derivation cohort (n=190,264)**	**Validation cohort (n=189,626)**	**p-value**
Median age (IQR)	54 (33-71)	54 (33-71)	.49
Male sex, n (%)	115,478 (60.7%)	115,284 (60.8%)	.53
Comorbidities, n (%)			
Congestive heart failure	6,710 (3.5%)	6,568 (3.5%)	.56
Cirrhosis	1,649 (0.9%)	1,708 (0.9%)	.29
COPD	12,520 (6.6%)	12,682 (6.7%)	.72
Diabetes	25,393 (13.3%)	25,260 (13.3%)	.55
Hypertension	61,611 (32.4%)	61,200 (32.3%)	.78
Smoking	38,118 (20.0%)	37,962 (20.0)	.61
End stage renal disease	3,166 (1.7%)	3,232 (1.7%)	.42
Median ISS (IQR)	6 (4, 10)	6 (4, 10)	.62
Trauma Center Criteria, n (%)			
GCS $\leq$ 13	12,034 (6.3%)	12,045 (6.4%)	.82
SBP $\leq$ 90	3,142 (1.7%)	3,200 (1.7%)	.63
Chest wall instability or deformity	565 (0.3%)	577 (0.3%)	.46
Penetrating injuries	8,859 (4.7%)	8,772 (4.6%)	.72
Pelvic fracture	1,176 (0.6%)	1,167 (0.6%)	.62
High risk blunt mechanism	12,922 (6.8%)	13,075 (6.9%)	.58

IQR: interquartile range, ISS: injury severity score, COPD: chronic obstructive pulmonary disease, GCS: Glasgow Coma Scale, SBP: systolic blood pressure

© 2022 Gutierrez A et al. *JAMA Network Open.*
